# Cord–Age–Gender Connections Shape the Pathophysiology of Multiple Sclerosis Progressive Forms

**DOI:** 10.3390/ijms20205103

**Published:** 2019-10-15

**Authors:** Serge Nataf

**Affiliations:** 1 Bank of Tissues and Cells, Lyon University Hospital (Hospices Civils de Lyon), 69003 Lyon, France; serge.nataf@inserm.fr; Tel.: +33-4-72-11-76-67; Fax: +33-4-72-11-96-49; 2 CarMeN Laboratory, INSERM 1060, INRA 1397, INSA Lyon, 69621 Villeurbanne, France; 3 Lyon-Est School of Medicine, University Claude Bernard Lyon-1, F-69000 Lyon, France

There is increasing evidence that sex hormones, aging, and the occurrence of spinal cord (SC) tissue alterations exert combined effects on the development and outcome of multiple sclerosis (MS) progressive forms. This view is best exemplified in patients suffering from primary progressive MS (PPMS). Indeed, a well-known yet frequently neglected feature of PPMS is a 1:1 sex ratio, which contrasts with the 3 female to 1 male ratio observed in relapsing remitting MS (RRMS) [1]. Accordingly, in patients with a radiologically isolated syndrome, being a male is a risk factor to develop PPMS [2]. Along this line, it has been established for a long time that the mean age of onset in PPMS varies between 40 and 50 years, which, on average, corresponds to a 10 years delay compared to RRMS [3]. Finally, the use of increasingly sophisticated magnetic resonance imaging (MRI) technologies has allowed one to establish two important points with regard to the links between PPMS and spinal cord tissue alterations. First, in patients with an established diagnostic of PPMS, SC atrophy and/or SC lesion load were shown to more reliably predict or reflect clinical disability, compared to their brain counterparts [4,5]. Second, in patients with a radiologically isolated syndrome, SC atrophy and/or SC lesion load were found to be early markers of progressive MS onset [2,6].

While such findings point to a connection between spinal cord, age, and gender in PPMS pathophysiology, other arguments indicate that, to some extent, this view might be extrapolated to secondary progressive MS (SPMS). Thus, irrespective of MS clinical forms, aging is a major risk factor of disease progression [7] and clinical progression in SPMS patients occurs at similar ages or even later compared to PPMS patients [8]. Also, with regard to gender, male RRMS patients are at higher risks for developing clinical worsening [9] or converting to SPMS [10]. Finally, compared to RRMS patients, spinal cord tissue alterations (including lesion load and atrophy) are more pronounced is SPMS patients [4,6,11] and may predict neurological disability [12].

However, when considering a pathophysiological connection between cord, age, and gender in MS progressive forms, one would expect that, in PPMS and SPMS, spinal cord lesions would exhibit more pronounced signs of inflammation, compared to brain lesions. On the contrary, paradoxically, spinal cords in progressive MS patients exhibit a lower percentage of active plaques and a higher percentage of chronic inactive plaques, compared to brains [13,14]. We would like here to argue that such an apparent discrepancy between neuropathological and clinico-radiological findings might be explained by the possible incompleteness of our current neuropathological classification regarding plaque activity. One should keep in mind that such a classification essentially relies on the general principle that inactive plaques are characterized by a lack of myelin-laden macrophages/microglia, whether within the plaque itself or in the periplaque area. This implies that astrocytes, the predominant glial cell type in chronic inactive plaques, are merely scar-associated astrocytes that are hence not “active”. Several works challenge this view. In particular, we demonstrated that in MS spinal cords, myelin-laden macrophages/microglia could be observed only exceptionally in large periplaque areas otherwise characterized by an extensive astrocytosis, partial demyelination, and a TGF-beta 1 molecular signature [15,16]. Moreover, astrocytes in inactive plaques were found to overexpress JAG1 (Jagged 1) [17,18], a TGFB1-induced astrocyte-expressed ligand that triggers the oligodendrogenesis-inhibiting Notch signaling pathway [18,19]. Altogether, these data suggest that “inactivity” with regard to the presence of myelin-laden macrophages/microglia does not equal “inactivity” regarding astrocytosis and, possibly, the astrocyte-driven progression of tissue alterations. 

Overall, future studies are needed to clarify the links between age, gender, and spinal cord tissue alterations in the pathophysiology of MS progressive forms. We suggest that such parameters are interconnected and that the aging-dependent decline in androgens and/or androgen precursors is amplifying a slowly evolving process of TGF-beta 1-mediated tissue remodeling that preferentially targets the spinal cord.
